# Monoclonal Antibody Against ROR1 Induces Apoptosis in Human Bladder Carcinoma Cells

**Published:** 2020

**Authors:** Ali-Ahmad Bayat, Niloufar Sadeghi, Ramina Fatemi, Mohammad Reza Nowroozi, Solmaz Ohadian Moghadam, Mohadeseh Borzuee, Amin Radmanesh, Mahmood Khodadoost, Ali Reza Sarrafzadeh, Omid Zarei, Hodjattallah Rabbani

**Affiliations:** 1. Monoclonal Antibody Research Center, Avicenna Research Institute, ACECR, Tehran, Iran; 2. Uro-Oncology Research Center, Tehran University of Medical Sciences, Tehran, Iran; 3. Legal Medicine Research Center, Legal Medicine Organization, Tehran, Iran; 4. Faculty of Traditional Medicine, Traditional Medicine and Materia Medica Research Center, Shahid Beheshti University of Medical Sciences, Tehran, Iran; 5. Department of Pathology, Khatam Al Anbia Hospital, Tehran, Iran; 6. Cellular and Molecular Research Center, Research Institute for Health Development, Kurdistan University of Medical Sciences, Sanandaj, Iran

**Keywords:** Bladder cancer, Flow cytometry, Monoclonal antibody, ROR1 protein

## Abstract

**Background:**

Receptor tyrosine kinase-like Orphan Receptor 1 (ROR1) is one of the promising cell surface antigens for targeting cancer cells. The aim of this study was to evaluate ROR1 cell surface expression in bladder cancer cells using a murine anti-ROR1 monoclonal antibody (mAb) called 5F1-B10 as well as investigate its potential in apoptosis induction.

**Methods:**

Expression of ROR1 in two human bladder cell lines, 5637 and EJ138, as well as a non-cancerous human cell line, Human Fetal Foreskin Fibroblast (HFFF), was examined by flow cytometry and immunocytochemistry. Immunohistochemical staining of cancer and normal bladder tissues was also performed.

**Results:**

The flow cytometry results showed that 5F1-B10 mAb could recognize ROR1 molecules in 86.1% and 45.6% of 5637 and EJ138 cells, respectively. The expression level of ROR1 was 5.49% in HFFF cells. The immunocytochemistry and immunohistochemistry staining results also confirmed the presence of ROR1 on the surface of both bladder cancer cells and tissues, respectively. The obtained data from apoptosis assay demonstrated that 5F1-B10 mAb could induce apoptosis in both 5637 and EJ138 cell lines.

**Conclusion:**

Taken together, our finding indicates the role of ROR1 in bladder cancer cell survival and suggests this receptor might be a promising target for developing novel therapeutic agents against bladder carcinoma.

## Introduction

Bladder cancer is the 10^th^ most common cancer with nearly 549,000 new cases and 200,000 deaths annually in both men and women worldwide. The incidence and mortality rates in men are four times higher than women ^1^. A wide range of risk factors have been associated with bladder cancer development as Cumberbatch *et al* reported ^2^. Regardless of different therapeutic procedures for treatment of bladder cancer ^3–6^, it is the 13^th^ cause of cancer death worldwide ^1^, indicating the necessity of developing novel therapeutic strategies.

The targeted therapy-based approaches attempt to find more effective therapeutics with minimal side effects towards the normal cells ^7^. In this regard, specific monoclonal Antibodies (mAbs) are well accepted therapeutic tools applicable in such targeted therapy strategies from the past to the present. There are several examples of successful therapeutic anti-cancer mAbs in the market ^8^ as well as in clinical trial studies ^9^. Selection of cancer specific and cell surface targets are key points to obtain effective antibodies. Receptor tyrosine kinase-like Orphan Receptor 1 (ROR1) is one of the promising cell surface antigens for targeting cancer cells by monoclonal antibodies ^10^.

ROR1 which is known as a diagnostic and prognostic biomarker in cancer patients ^11–13^ is a transmembrane glycoprotein member of Receptor Tyrosine Kinase (RTK) superfamily ^14^. ROR1 encoding gene is located on chromosome 1p31-p3 and produces a ∼105 *kDa* protein with 937 amino acids, that structurally consist of extracellular parts, a transmembrane segment, and intercellular regions ^15^. The extracellular parts of human ROR1 possess different parts including immunoglobulin-like, cysteine-rich, frizzled and kringle domains ^16^. Although the kirngle domain is more specific to ROR1 compared to other RTKs, but targeting CRD domains as ligand binding site by monoclonal antibody is preferred ^17–19^.

The roles of ROR1 expression and activation in normal embryonic and fetal development have extensively been documented ^20,21^. Besides these physiological roles, its aberrant expression in different types of cancers from hematological malignancies to solid tumors has been shown ^22–24^. Moreover, ROR1 expression is in line with cancer cell progression, invasion and metastasis, induction of Epithelial-Mesenchymal Transition (EMT), and drug resistance ^25–28^.

Considering the importance of ROR1 as a target in cancer and lack of comprehensive studies for cell surface expression of ROR1 in bladder cancer ^22^, an attempt was made to evaluate the cell surface ROR1 expression in bladder cancer cells using our previously produced anti-ROR1 monoclonal antibody targeting CRD domain ^29^ and its potential role in apoptosis induction in these cancer cells.

## Materials and Methods

### Cell culture

Two human bladder carcinoma cell lines, EJ138 and 5637, and Human Fetal Foreskin Fibroblast (HFFF) were obtained from National Cell Bank of Iran (Pasteur institute, Tehran, Iran) and cultured in RPMI-1640 medium (Gibco, Grand Island, NY, USA), supplemented with 10% fetal bovine serum (FBS) (GIBCO Invitrogen, USA) plus penicillin (100 *U/ml*)/streptomycin (100 *μg/ml*) and were incubated at 37°*C* under humidified atmosphere containing 5% CO_2_.

### ROR1 cell surface expression by flow cytometry

To detect the ROR1 cell surface expression, the cells were cultured in tissue culture flask until reaching 70–80% confluency. The cells were detached by citrate buffer and washed three times with cold Phosphate Buffered Saline (PBS) and transferred to flow cytometry tubes. Blocking was performed using 5% sheep serum for 30 *min* at 4°*C*. The cells were incubated with either anti-ROR1 monoclonal antibody clone 5F1-B10 at 10 *μg/ml* concentrations (Padza Co., Iran) ^29^ or isotype control mAb for 1 *hr* at 4°*C*. After three times of washing, the FITC conjugated sheep anti-mouse immunoglobulin (Padza Co., Iran) (1:50) was added to the cells and incubation continued for 45 *min* at 4°*C* in a dark place. The cells were finally washed and subjected to PAS III flow cytometer (Partec GmbH, Germany). The data were analyzed using FlowJo software, version 10. The average FITC intensities were calculated by multiplication of Mean Fluorescence Intensity (MFI) to the Percentage of Positive cells (POP) (MFI×POP).

### Immunocytochemistry (ICC)

Immunocytochemistry (ICC) was performed to assess the ability of 5F1-B10 antibody to recognize ROR1 in bladder cancer cells. The cultured cells were harvested using 0.25% trypsin with 0.1% EDTA (Gibco), seeded at a density of 2×10^4^ cells on an 8-well cover slip (Marienfeld GmbH, Lauda-Königshofen, Germany) and incubated at 37°*C* as described above. After overnight incubation, the medium was removed and the cells were fixed using acetone (Pre-incubated at −20°*C*) for 2 *min*. The process was followed by incubating at 4°*C* for 30 *min*. The slides were washed three times (3×3 *min*) with Tris-Buffered Saline (TBS) (pH=7.4) containing 0.1% Bovine Serum Albumin (BSA). The slides were blocked with 10% sheep serum in TBS containing 1% BSA (1% TBS-BSA) for 30 *min* at Room Temperature (RT). Subsequently, the slides were incubated with 10 *μg/ml* of 5F1-B10 antibody or isotype control mAb, diluted with 1% TBS-BSA. The incubation continued at RT for 60 *min*, and the slides were washed with TBS and TBS-BSA for three times.

FITC-conjugated sheep anti-mouse immunoglobulin (Padza Co., Iran) was added to the slides at 1:100 dilution, and they were incubated at RT for additional 45 *min*. The slides were subjected for 3 times of washing. To counterstain the cell nuclei, 4′,6-diamidino-2-phenylindole dihydrochloride (DAPI) (Calbiochem, USA) at 1 *μg/ml* concentration was used for 10 *min*. Finally, the slides were washed with TBS, mounted in 50% TBS-glycerol and examined under a fluorescent microscope (Olympus, Tokyo, Japan).

### Apoptosis induction

Annexin V-FITC and Propidium Iodide (PI) (BD Biosciences, San Jose, CA) were used to evaluate the apoptosis induction in bladder cancer cells by flow cytometry using anti-ROR1 monoclonal antibody (clone 5F1-B10).

Briefly, the cells were cultured in 24-well plates (1.5×10^4^ cells/well) and incubated for 6 and 12 *hr* with 10 *μg/ml* 5F1-B10 or isotype control mAb. After incubation, the cells were harvested and washed with cold PBS followed by staining with annexin V-FITC and PI. Incubation was performed at RT at a dark place for 15 *min*. The percentage of apoptotic cells as well as cell viability was measured by Partec PAS III flow cytometer (Partec GmbH, Germany).

### Immunohistochemistry (IHC)

Immunohistochemical analysis was performed on Formalin-Fixed Paraffin-Embedded (FFPE) human bladder carcinoma (Imam Khomeini Hospital, Tehran, Iran) and normal bladder tissues (National Forensics Organization, Tehran, Iran(. Briefly, 4 *μm* of thick tissue sections were deparaffinized with xylene, then rehydrated in decreasing concentrations of ethanol. Antigen retrieval was done in citrate buffer (10 *mM*, pH=6) at 94°*C* for 30 *min* in water bath. After three times of washing by 0.1% TBS-BSA (pH=7.4), the endogenous peroxidase activity was quenched by addition of 3% H_2_O_2_ in TBS for 15 *min*. Nonspecific binding was blocked with 5% goat serum in 2.5% TBS- BSA for 30 *min* followed by incubation with 10 *μg/ml* anti-ROR1 mAb in 2.5% TBS- BSA for 60 *min* at RT. Next, the sections were washed and incubated with EnVision detection system (BioGenex, United States) for 30 *min* at RT. Color was developed using 3, 3′-diaminobenzidine (DAB) substrate solution (BioGenex) and the slides were counterstained with Mayer’s hematoxylin. The slides were then washed with deionized water, dehydrated with graded ethanol, mounted with Entellan (Merck, Germany) ^30^ and examined under a fluorescent microscope (Olympus, Tokyo, Japan). Anti-beta actin and mouse IgG isotype control antibodies were also employed as controls.

## Results

**Figure 1.**
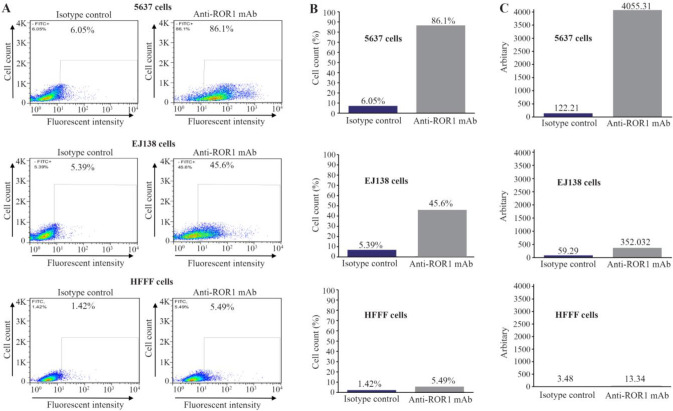
Reactivity of anti-ROR1 monoclonal antibody clone 5F1-B10 to bladder cancer and normal cell lines using flow cytometry. Left panel: A) 5F1-B10 could react with ROR1 in 86.1% of 5637 and 45.6% of EJ138 cells, compared to HFFF cell (5.49%) as a normal sample. The values for isotype controls in all three cell lines have also illustrated. Middle panel: B) The same results illustrated as bars for better visualization. Right panel: C) The average FITC intensities were calculated through multiplying the mean fluorescence intensity by percentage of positivity (MFI×POP).

**Figure 2.**
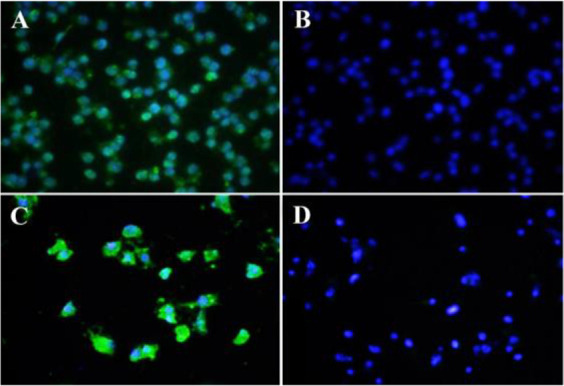
Immunocytochemistry (ICC) assay on bladder carcinoma cell lines. Mouse monoclonal anti-ROR1 antibody 5F1-B10 was used as a primary antibody and FITC-conjugated sheep anti-mouse antibody as secondary antibody (Green). DAPI was used for counterstaining the nucleus (Blue). A (5637 cells), C (EJ138 cells), mouse IgG isotype controls (B and D).

**Figure 3.**
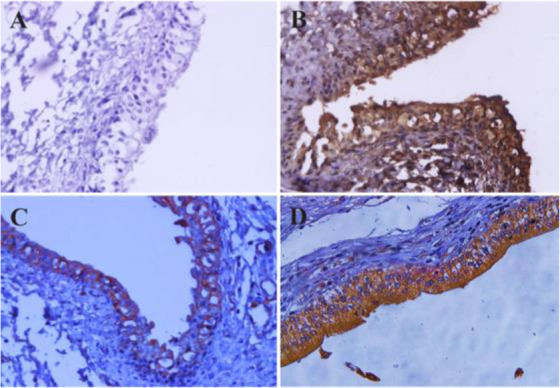
Detection of ROR1 expression in normal and bladder cancer tissues by immunohistochemistry (IHC). Anti-ROR1 mouse monoclonal antibody (5F1-B10), mouse IgG isotype, and anti-beta actin antibodies were used as primary antibody. EnVision detection system (BioGenex, United States) was employed for signal detection and Mayer’s hematoxylin was used for counterstaining. A) Bladder carcinoma tissue and isotype control antibody, B) Bladder carcinoma tissue and anti-beta actin antibody, C) Normal bladder tissue and 5F1-B10 antibody, D) Bladder carcinoma tissue and 5F1-B10 antibody (Original magnification, ×50).

The expression of ROR1 in 5637, EJ138 and HFFF cells was investigated by flow cytometry. As it is shown in figure 1, the 5F1-B10 mAb recognized ROR1 molecules in 86.1% (5637 cells) and 45.6% (EJ138 cells) while the expression of ROR1 in HFFF as a negative control cell was 5.49%. For calculating the average of FITC intensity, Mean Fluorescence Intensity (MFI) was multiplied by Percentage of Positive cells (POP) (MFI×POP). The arbitrary values were 4055.31 (5637 cells), 352.32 (EJ138 cells) and 13.34 (HFFF) (Figure 1, Table 1). The expression of ROR1 in both bladder cancer cell lines was confirmed by immunofluorescence staining (Figure 2). Immunohistochemical staining of human bladder carcinoma and normal bladder tissues also revealed a high level of ROR1 expression in the bladder carcinoma tissue in comparison with normal tissue (Figure 3).

**Table 1.** Flow cytometry on bladder cancer and normal cell lines

**Cell line**	**Antibody**	MFI ^**^	POP ^***^	**MFI×POP**
**5637**				
	Anti-ROR1 mAb^*^	47.1	86.1	4055.31
	Isotype control	20.2	6.05	122.21
**EJ138**				
	Anti-ROR1 mAb	7.72	45.6	352.03
	Isotype control	11	5.39	59.29
**HFFF**				
	Anti-ROR1 mAb	2.43	5.49	13.34
	Isotype control	2.45	1.42	3.48

* Monoclonal antibody.

** Mean fluorescence intensity.

*** Percentage of positive cells.

**Figure 4.**
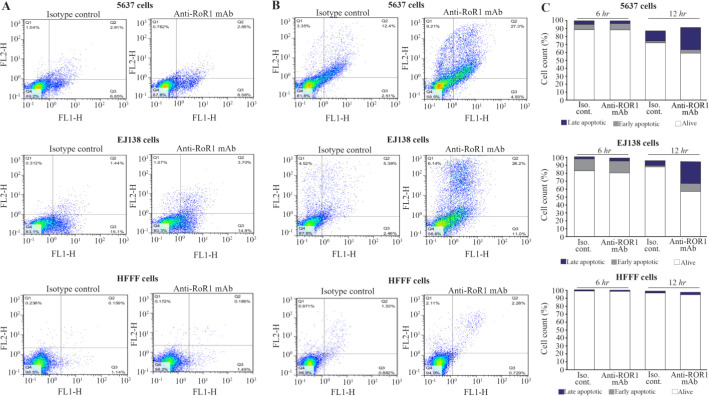
A flow cytometric apoptosis assay was performed by anti-ROR1 mouse monoclonal antibody (5F1-B10) on bladder cancer cell lines for 6 and 12 *hr*. Left panel: A) The antibody could induce apoptosis (Early and late apoptosis) in 5637 and EJ138 cells after 6 *hr*. Middle panel: B) The same experiment after 12 *hr*. Left panel: C) For better visualization of results a bar graph was drawn. The percentage of viable cells after 6 and 12 *hr* treatment for 5637 cells were 87.8% and 58.5% and for EJ138 were 80.3% and 56.6%, respectively. The viability of cells remains almost unchanged in both lines after 12 *hr* of induction. The anti-ROR1 antibody also did not induce apoptosis in HFFF cells as a normal cell line (The lower panels in A, B and C).

To evaluate the apoptosis induction of 5F1-B10 mAb, the 5637, EJ138, and HFFF cells were treated with 10 *μg/ml* of mAb for 6 and 12 *hr*. The results of 6 *hr* of incubation demonstrated that 5F1-B10 mAb could induce apoptosis in 5637 cell line with 8.58% and 2.85% of the cells for early and late apoptosis, respectively. The values for 12 *hr* incubation were 4.93% and 27.3% for early and late apoptosis, respectively. The results of 6 *hr* of incubation demonstrated that 5F1-B10 mAb could induce apoptosis in EJ138 cell line with 14.9 and 3.7% of the cells for early and late apoptosis, respectively. The values for 12 *hr* of incubation were 11 and 26.2% for early and late apoptosis, respectively. In the case of HFFF cells, the apoptosis induction for early and late apoptosis was 1.49 and 0.189% after 6 *hr* and 0.729 and 2.28% after 12 *hr* of incubations. The isotype control mAb could not induce significant apoptosis in all examined cells (Figure 4).

## Discussion

The involvement of ROR1 in tumorigenesis of many solid tumors and hematological malignancies has already been documented ^23,24,27,31^. So far, no functional role of ROR1 in bladder carcinoma cell lines has been reported. In the current work, the presence of cell surface ROR1 in bladder cancer cells was observed by flow cytometry using our previously produced anti-ROR1 antibody ^29^. To the best of our knowledge, this is the first report regarding functional study of ROR1 in bladder cancer by means of flow cytometry. Our results indicate that 5F1-B10 mAb recognizes ROR1 molecules in 86.1 and 45.6% of 5637 and EJ138 cells, respectively. ROR1 expression in bladder cancer cells (5637 and EJ138) was also examined by immunocytochemistry verifying the flow cytometry results (Figure 2).

### ROR1 diagnostic values in bladder cancer

The current diagnostic methods for bladder carcinoma include an invasive method of cystoscopy and non-invasive but low sensitive method of urine cytology. In 2002, Zhang *et al* reported the expression of ROR1 in primary bladder tumors by immunoblotting and formalin-fixed, paraffin-embedded tissue microarray assays ^22^. Our findings are in line with the report of Zhang *et al* suggesting ROR1 as a diagnostic biomarker in blabber cancer patients. Therefore, a noninvasive diagnostic as well as a monitoring method using urine samples from patients with bladder cancer employing anti-ROR1 antibody in flow cytometry technique is recommended. Furthermore, the results from immunohistochemistry clearly show the significance of using anti-ROR1 antibody as an additional diagnostic marker in patients with bladder carcinoma.

### Functional significance of ROR1 in bladder cancer

In flow cytometry assays, MFI was multiplied by the percentage of reactivity for obtaining an arbitrary value (Table 1) for estimating the number of receptors on the tumor cells ^32^. There was a significant difference in the number of ROR1 receptors on both cell lines. Careful analysis of apoptosis assays indicates almost an equal viability in both cell lines while treated with equal amount of antibody. The apoptosis induction of 5F1-B10 mAb in bladder cancer cells was evaluated. As it is understandable from figure 4, for the treated cells with 5F10-B10 mAb after 6 *hr*, no significant impact was observed while in 12 *hr* of incubation, the viability of the cells was reduced to 58.5% and 56.6% for 5637 and EJ138 cells with the anti-ROR1 mAb, respectively. This shows almost an equal number of both cell lines which underwent apoptosis (Figure 4). This is an interesting finding hence regardless of differences in ROR1 expression levels among 5637 and EJ138 cells (Figure 1), the percentages of apoptotic cells are the same. The current result shows ROR1, even in low amount of expression, plays a critical role in bladder cancer cell surviving and this is in agreement with previously available reports concerning the ROR1 importance as a survival factor as well as therapeutic target for other malignancies ^33^. The results from this part are in line with other reports emphasizing usefulness of ROR1 targeting by monoclonal antibody as a cancer therapy strategy ^24,34,35^.

ROR1 is not a bladder cancer specific marker and expressed in many other cancers ^17,22,29,33,36,37^. Targeting by therapeutic agents specially monoclonal antibodies might be considered as an option for treatment of bladder cancer. One should bear in mind the complications and side effects induced by employing the common conventional bladder cancer treatment strategies such as surgery, radiation therapy, intravesical chemotherapy (especially Mitomycin C), and intravesical immunotherapy (especially bacillus Calmette-Guerin, BCG).

Cancer targeted therapy using specific monoclonal antibodies reduces most of such complications owing its specific reactivity to corresponding target ^38^. There are some monoclonal antibodies against different targets of bladder cancers in preclinical and clinical trial phases ^39–41^, which highlights the importance of using mAbs in bladder cancer treatment.

In the case of monoclonal antibody against ROR1, at least there is a mAb (called Cirmtuzumab) in clinical trials which is an evidence for introducing ROR1 as a cancer target ^42,43^. Although the effectiveness of Cirmtuzumab against bladder cancer has not yet been evaluated but the current study might support employing Cirmtuzumab in bladder cancer treatment.

The current treatment of bladder carcinomas using FDA-approved monoclonal antibodies gives rise to a maximum efficacy of 29% in patients ^44^. The targets for such treatments include PD-L1 and PD-1 which may not be a survival factor in bladder cancer cells. Our ROR1 antibody alone is also inducing apoptosis of bladder cancer cells. This implies that ROR1 might be the only survival factor in bladder carcinoma and this could be the reason for not identifying so many targets for targeted therapy of this cancer. In experiments performed by Yin *et al* using a mouse model of ovarian carcinoma, the efficacy of ROR1 mAb was evaluated. In their experiments, they used a fully human-mouse chimeric IgG antibody which lacks any mouse FC part, ruling out any possible role of ADCC or CDC ^45^. The conditions for our *in vitro* apoptosis assay also lack any effector cells (T cells or NK cells) which highlights the ROR1 function could easily be affected by trace amount of antibody. This may indicate there is no alternative signaling pathway to compensate lack of ROR1 in cell survival of bladder cancer cells. This notion needs further investigations with special focus on the nature of bladder cancer cells.

## Conclusion

Taken together, our findings indicate the critical role of ROR1 in bladder cancer cell survival and suggest this receptor as a promising target for development of novel therapeutic agents against bladder cancer cells. Further *in vivo* studies is needed using a mouse model of human bladder carcinoma lacking cellular immunity (*e.g.* nude mouse) to apply only anti-ROR1 monoclonal antibody to draw such conclusion.
